# Fire Extinguishing Appliances

**Published:** 1911-05-13

**Authors:** 


### FIRE EXTINGUISHING APPLIANCES.

There are many small establishments in the kingdom of the Cottage Hospital and Convalescent Home class which, by reason of their gradual growth from what was in all probability at first an ordinary house, have not had great attention paid to their fire-resisting construction. To reconstruct them on modern lines is out of the question, but it is quite possible that with a minimum of expenditure they may be equipped with appliances to combat fire and that successfully—and, in fact, owing to the reduction on premiums allowed by insurance companies, such appliances would prove an economy.

We must first premise that alternative means of escape will be provided from each part of the building, so that if one line of retreat is cut off, another is available. Further, that disused fireplaces are solidly built up: these often occur in cases where probably a partition has been removed in order to obtain a larger apartment, and are sometimes covered with some light woodwork to carry the wallpaper on. This is dangerous, and the Glasgow authorities, among others, realising the danger, have a law to the effect that every fireplace opening which contains no grate or stove shall be stopped up with incombustible materials, such as brickwork, and the chimney top shall likewise be plugged with fire-resisting material.

The usual fire-extinguishing appliances for a small establishment may be divided into two classes, namely, water appliances and chemical appliances. Under the former head will come hydrants, pumps, and hand buckets. Hydrants are attached to a water main and the water conducted by hose sufficient in length to reach any point of the building. This hose is made of selected flax yarn and varies in diameter from 1½ in. to 2½ in. for a small installation, finishing with a gunmetal nozzle. The water supply for a hydrant system will often be found insufficient in power to rise to the heights required and recourse must be had to an accelerator or force pump. In larger hospitals this might be a steam engine, but in small country homes a "manual" will generally be found sufficient—such as has been installed at the Children's Homes, Sidcup. The mechanism of this machine is such that by suction it takes up the water and throws it to a considerable height—about fifty feet—in one or more jets; the labour is by hand pumping and may be worked by two or more persons, depending on the size of the machine. The cost of a small manual fire engine might vary between £20 and £40. (Note.—The Sidcup manual is a fire engine suitable for a brigade, listed at £120 or so, and twenty to twenty-four boys can be put on the pumping handles.)

In cases where proper fire hydrants are not deemed advisable, and even where they are used, but it is thought desirable to augment them, a useful appliance may be had in the form of a portable hand pump fire apparatus. This is a very popular form of fire extinguisher, as may be evinced from the fact that one particular hospital has no less than eighty-three of them in use. And it was reported by the Captain of the London Fire Brigade that 2,540 out of 4,199 fires were extinguished by this class of hand pump in one year. It is portable, simple to use and useful for putting out fires in their elementary stages, and only costs about five guineas. This pump may be had with greater power at a cost of about ten pounds—it will throw twelve gallons per minute to a height of forty feet, and is only twenty inches high by eighteen inches diameter. Of course, with these hand pumps it is necessary that someone should be replenishing the water tank while pumping is being done, for though the last-named pump is capable of throwing twelve gallons a minute, the tank or pail only holds seven gallons.

A sight that gives a nervous person confidence is that of a row of water buckets on entering an hotel or other public place, and no doubt the same sensation might apply in a hospital building, but the buckets filled with water are not altogether so hygienic as might be desired. A tank is now on the market which has a lid to it, and this lid on being lifted off discloses a bucket full of water ready to be lifted out. When this top one has been lifted out another is seen ready full of water, and so on to the number of seven or more. This "cabinet" of buckets has the effect of keeping the water clean and sweet and the lid reduces evaporation to a minimum; rather an important factor in actuality. The cost of a nest of buckets such as before described might be about seven pounds.

In the second class, namely, chemical fire-extinguishing appliances, we have machines varying in cost from 25s. to £125, but, generally speaking, all are based on one idea, namely, that the appliances are charged with a solution of bicarbonate of soda and a bottle of sulphuric acid. In being brought into use the acid bottle is broken, when an immense quantity of carbonic acid gas is formed, and an effervescing fluid of great extinguishing power is discharged at a velocity sufficient to carry it a distance of forty feet or more from the nozzle.

In buildings of the smaller hospital type there are sometimes attics from which, in case of fire, servants would have to escape, it being customary, so far as possible, not to have patients on the higher floors. Where it is inconvenient to have gangway or ladder escapes a useful appliance may be found in the "chute" escape, which consists of a wrought-iron frame and tube of strong canvas. When it is required for use a window is opened, the iron frame placed on the sill and by snap hooks secured to two eye bolts in the floor; then the canvas tube is thrown out of the window and falls to the ground. If no persons are available on the ground to hold the canvas end well away, the first person to descend may regulate his speed by pressing knees and elbows against the side of the tube.

A type of chute escape has been recommended by H.M. Commissioners in Lunacy for all asylums owing to its safety and simplicity. The cost of an escape of this type for a fifteen-feet height is about £5, and may be had in proportionate prices up to a length of seventy-six feet, which would cost about £16 10s.

